# A Wireless Fatigue Monitoring System Utilizing a Bio-Inspired Tree Ring Data Tracking Technique

**DOI:** 10.3390/s140304364

**Published:** 2014-03-05

**Authors:** Shi Bai, Xuan Li, Zhaohui Xie, Zhi Zhou, Jinping Ou

**Affiliations:** 1 School of Civil Engineering, Harbin Institute of Technology, Harbin 150090, China; E-Mails: stone3214@163.com (S.B.); oujinping@dlut.edu.cn (J.O.); 2 School of Civil Engineering, Dalian University of Technology, Dalian 116024, China; E-Mail: milanlx1988@hotmail.com; 3 School of Software Technology, Dalian University of Technology, Dalian 116024, China; E-Mail: xie.zhao.hui@foxmail.com

**Keywords:** fatigue, structural health monitoring (SHM), rain-flow counting method, data tracking of tree rings, Digital Signal Processing (DSP), wireless sensor, PVDF

## Abstract

Fatigue, a hot scientific research topic for centuries, can trigger sudden failure of critical structures such as aircraft and railway systems, resulting in enormous casualties as well as economic losses. The fatigue life of certain structures is intrinsically random and few monitoring techniques are capable of tracking the full life-cycle fatigue damage. In this paper, a novel *in-situ* wireless real-time fatigue monitoring system using a bio-inspired tree ring data tracking technique is proposed. The general framework, methodology, and verification of this intelligent system are discussed in details. The rain-flow counting (RFC) method is adopted as the core algorithm which quantifies fatigue damages, and Digital Signal Processing (DSP) is introduced as the core module for data collection and analysis. Laboratory test results based on strain gauges and polyvinylidene fluoride (PVDF) sensors have shown that the developed intelligent system can provide a reliable quick feedback and early warning of fatigue failure. With the merits of low cost, high accuracy and great reliability, the developed wireless fatigue sensing system can be further applied to mechanical engineering, civil infrastructures, transportation systems, aerospace engineering, *etc*.

## 1. Introduction

Fatigue is a process of progressive local permanent structural change occurring in a material under cyclic loading. These permanent structural changes may culminate in cracks, which may further result in a failure of the entire structure after a certain number of additional loading cycles. Fatigue failures are often catastrophic since they usually occur without any warning, which may cause the loss of lives as well as significant property damage. Fatigue is one of the major factors for the failure of mechanical structures and members [1]. Statistics has shown that fatigue damages are responsible for 50% to 90% of the failures of mechanical structures [2,3]. Thus, the prediction of fatigue life of structures has been one of the dominant issues for structures exposed to repeated cyclic loading in various applications such as machines, civil infrastructures, transportation systems, aerospace structures and energy related structures. However, due to the complexity and uncertainty of service environments as well as multiple damage mechanisms, an accurate estimation of the remaining fatigue life is hard to achieve for aging infrastructures under cyclic loading.

Structural Health Monitoring (SHM) is the basis of the life-cycle performance-based design approach, which helps improve the safety, durability, serviceability and sustainability of a structure for long-term operation [4]. A structure with a SHM system can be considered as a full-scale experimental model and system [5]. The rapid development of the SHM technique provides a potential solution for the fatigue life prediction. Fatigue damage, known as a stochastic phenomenon, makes online monitoring/sensing a significant need for a reliable estimate of the remaining fatigue life [6]. To meet this need, various sensing technologies have been developed in last century for fatigue damage monitoring and structural failure analysis, including acoustic emission [7,8], ultrasound [9,10] and eddy currents [11,12], fatigue life gauges [13], electrochemical fatigue sensors (EFSs) [14,15] and thermal imaging techniques [16].

The acoustic emission technique [7,8] takes advantage of the benefits of the correlation between the detected signals (e.g., the acoustic emission counts, the peak amplitudes and the energy) and the fatigue crack initiation. If high sensitivity for crack detection is achieved, this technique is able to capture the initiation of microstructural fatigue. However, the sensitivity of this method is limited by the signal-to-noise ratio. In noisy environments, the acoustic emission may not work well, taking account of the difficulty of separating the signal from noise. In an effort to detect fatigue crack in noisy environments, the ultrasonic sensing technique is recommended [9,10]. For this sensing technique, high-frequency ultrasonic pulses emitted by an ultrasonic sensor travel through the specimen carrying the structural behavior information, and are received by the transducers at the other end. The ultrasonic sensing technique could distinguish small changes of the specimen during the early stages of fatigue damage, which may even not be able to be detected by an optical microscope. The ultrasonic sensing technique has been utilized to monitor the small scale microstructural fatigue damage evolution.

In addition, if the monitored structures are made up by conductive materials and only surface cracks are expected, the eddy current technique can also be employed [11,12]. The eddy current technique is based on the principles of electromagnetic induction, and it can detect the presence of faults through the affected eddy current flow patterns, which can be utilized to detect the evolution of small fatigue cracks. In the case that a high frequency fatigue response monitoring is required, the fatigue life gauge can be applied [13]. The fatigue life gauge is an electrical resistance-based fatigue strain sensor based on a concept similar to that of a foil strain gauge for normal loading conditions. Experimental work has demonstrated that the fatigue life gauge can provide stable high-frequency fatigue responses repetitively. However, the commonly used fatigue life gauge cannot cover the low strain cycles, and it also has the drawbacks of low durability and nonlinear effects.

Fatigue can be characterized by three parameters obtained from the SHM system: the number of cycles, the strain amplitude, and the state of stress. If the fatigue life at any given stress level and the number of cycles at the corresponding stress level are monitored, the aggregate life can be calculated using fatigue damage cumulative algorithms like the Palmgren–Miner linear rule [17]. Several efficient fatigue prediction methods have been developed based on the statistical measurements of the number of fatigue cycles, such as level cross counting, peak-valley value counting, simple range counting, rain-flow counting, and hysteresis loop counting.

Among these methods, the rain-flow counting method is the most widely applied one. The basic concept of rain-flow counting method was initially introduced by Matsuishi and Endo in 1968 to count the cycles or the half cycles of strain-time signals [18]. It was named for the way that rain runs off the roofs of pagodas. Counting is based on the stress-strain behavior of the material. The rain-flow counting was further improved by Downing [19] making the algorithm more convenient for practical fatigue design and analysis. A modified rain-flow algorithm accounting for the load sequence effect has also been developed [20], and the regenerated loading histories can yield similar crack growth results using fracture mechanics-based models [21]. Many efforts have been directed to make it more comprehensive and sophisticated. For example, Jiang [22] proposed an easily programmed recursive algorithm. Tian [23] developed an improved model of rain-flow for real-time counting in which rectification or modification of the stress-time history is no longer necessary before counting. This model was successfully applied to the data treatment of stress-spectra for high-speed Electric Multiple Units (EMUs). Xiao [24] enhanced the three-point rain-flow counting method and applied it to the evaluation of blasting damage. Carmen Castillo [25] replaced the commonly used records of wave heights employed in the design of marine structures with rain-flow matrices, and thus made an obvious improvement on the safety of the designed structures.

In this paper, an intelligent system for fatigue damage assessment and fatigue life prediction of in-service structures is developed based on the online real-time SHM data. The system is inspired by the concept of tree ring data tracking. The rings of semi-tropical trees can clearly record the growth features of trees, such as irreversible age gaining, boundaries between each year, and speed of growth under various environmental conditions, *etc*. Similar phenomena are found when comparing the fatigue feature parameters (strain amplitude, number of cycles and state of stress) to that of the tree-rings data tracking. Thus, supported by fatigue loading spectrum theory, electronics and microprocessor technologies and wireless transmission technology, the intelligent system is designed and integrated. Laboratory tests based on strain gauges and PVDF sensors were set up to investigate the properties of the system.

## 2. Operational Principle

### 2.1. Tree-Rings Data Tracking

Nature not only provides an information sourcebook of behavior, function, color and shape which can inspire visual design and invention [26], but acts as a school for materials science and its associated disciplines such as chemistry, biology, physics or engineering [27]. Conventional analysis of fatigue damage accumulation based on the whole strain course has disadvantages of high cost, complicated sensor implementation and system integration, making it hard to use in engineering applications. In combination with a counter based on Digital Signal Processing (DSP) technology, the bio-inspirited tree-rings data tracking method can record the real-time characteristics of fatigue behavior: the amplitude of strain, the number of cycles, and the stress state, potentially overcoming the disadvantages of the traditional fatigue prediction methods. The tree-rings data tracking method could record various environmental conditions; the DSP hardware technique, on the other hand, records, analyzes, and transmits the real-time characteristic data from the wireless fatigue meter, such as the amplitude of strain, the number of cycles and the stress state. With both the environmental conditions and the fatigue progressing, the analysis of fatigue damage accumulation and an early warning for potential structural fatigue failure can be estimated.

### 2.2. Rain-Flow Counting Method for Fatigue Data Tracking

**Figure 1.**
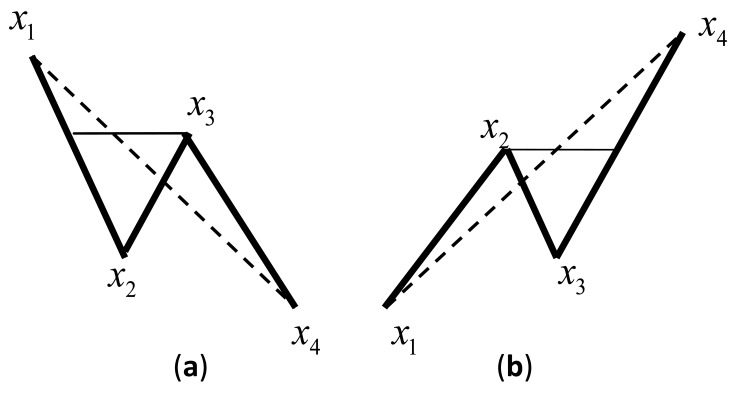
Criterion conditions of rain-flow counting method.

In this paper, four Peak-to-Valley values comparison algorithm was adopted as the rain-flow counting method. As illustrated in Figure 1, a cycle should be counted as long as either condition a or b (indicated by Equations (1) and (2)) occurs, and the amplitude and the mean values of the cycle are recorded accordingly. After removing the middle two values, another two values are taken out from the peak/valley values array (array X), then forming a new judgement of four peak-to-valley values:

(1) $$\text{Condition a}:\text{if}\;x_{1}\geq x_{2},\text{both}\;x_{1}\geq x_{3}\;\text{and}\;x_{2}\geq x_{4}$$

(2) $$\text{Condition b}:\text{if}\;x_{1}\leq x_{2},\text{both}\;x_{1}\leq x_{3}\;\text{and}\;x_{2}\leq x_{4}$$

## 3. System Design of the Intelligent System

### 3.1. System Integration

**Figure 2.**
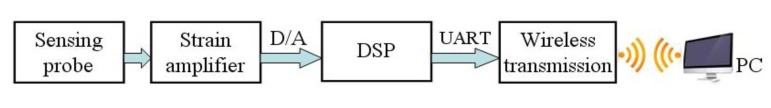
Wireless fatigue monitoring system.

Figure 2 shows the flow chart of the wireless fatigue monitoring system. The time history of the object's fatigue response is sensed by the sensing probe. The obtained data is transmitted to a strain amplifying processor, which achieves the digital-to-analog conversion to convert the time history into digital signal. The DSP module implemented with rain-flow counting method analyzes the signals, and the key parameters of fatigue features are acquired and saved in the DSP microchip and finally transmitted by the wireless transmitter module to the user PC. The fatigue damage is estimated using the acquired key parameters.

### 3.2. Wireless Communication and Networking

**Figure 3.**
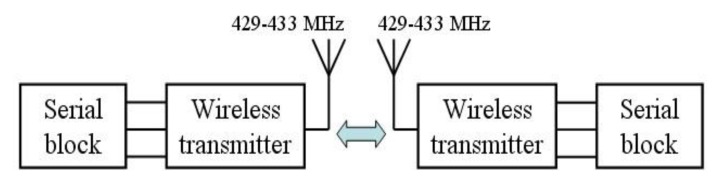
Point-to-point wireless serial communication.

Taking advantage of low price, good stability and linearity, excellent corrosion resistance and developed technology, the electrical resistance based strain gauge is used as the major sensing probe. The strain amplifying module amplifies, filters, and demodulates the strain gauge data, followed by data collection using DSP. A F28335 DSP microchip made by YanXu Company, Nanjing, China, is used in this study. The interface between DSP module and wireless transmission module is a serial communication. The wireless transmitter adopts STR-30 micro-power wireless transfer-block, in view of its advantages of remote transmitting power, long transmission distance (within the range of sight distance, antenna height >1.5 m, reliable transmission distance >800 m), low Bit Error Rate (BER), small-size, and low weight. Point-to-point wireless serial communication can help the wireless module work firmly and exactly, as shown in Figure 3.

### 3.3. Digital Signal Processing and Hardware Module Design

**Figure 4.**
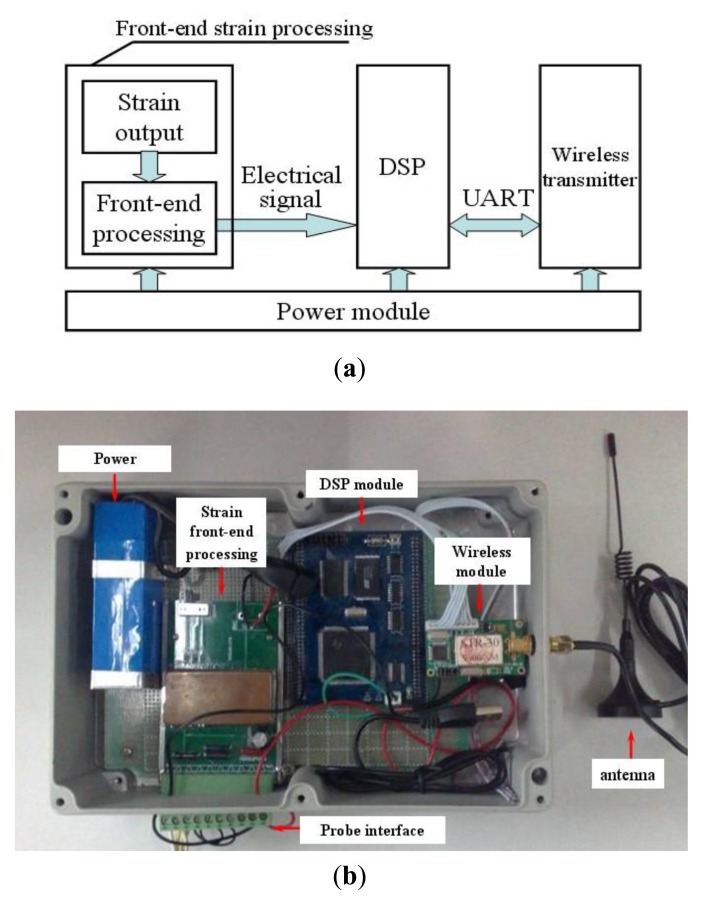
(**a**) The installation of hardware integration; (**b**) Photo of the system.

Figure 4 shows the details of the hardware design for the developed system including the front-end strain processor, the DSP, the wireless transmitter, and the power module. The power module consists of two components, an YSD-12-5 Li-ion battery and a B0503M-1W DC-DC power converter. The loop charging battery supplies a consistent voltage of 5 V, and has a capacitance of 9000 mAh. The DC-DC power converter is usually utilized in low power supply system containing distributed generation. The strain front-end processor requires a 5 V power supply, while the DSP and the wireless transmitter need 3.3 V power supplies. The voltage conversion can be realized by the DC-DC power module. Figure 4b shows a photo of the system.

## 4. Experiments and Discussion

### 4.1. Numerical Simulation

**Figure 5.**
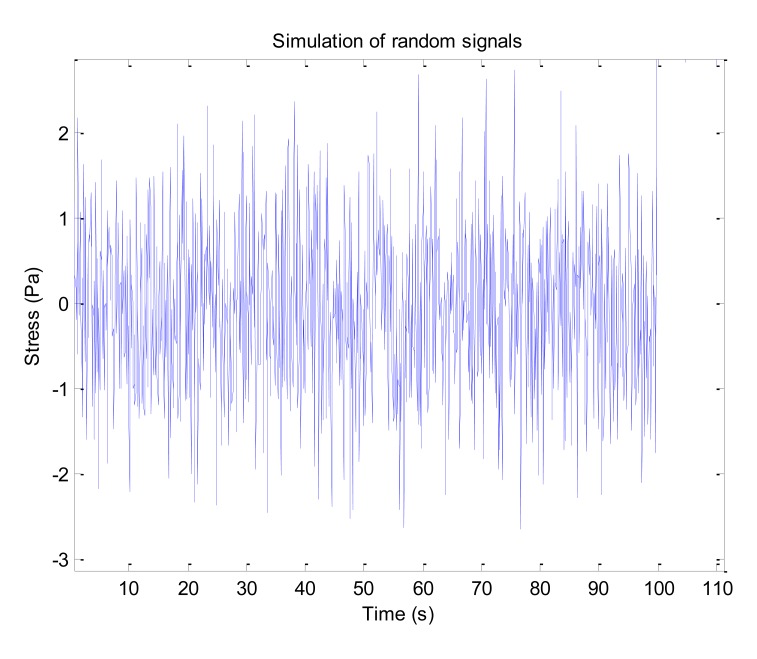
Numerical simulation of random signals.

**Figure 6.**
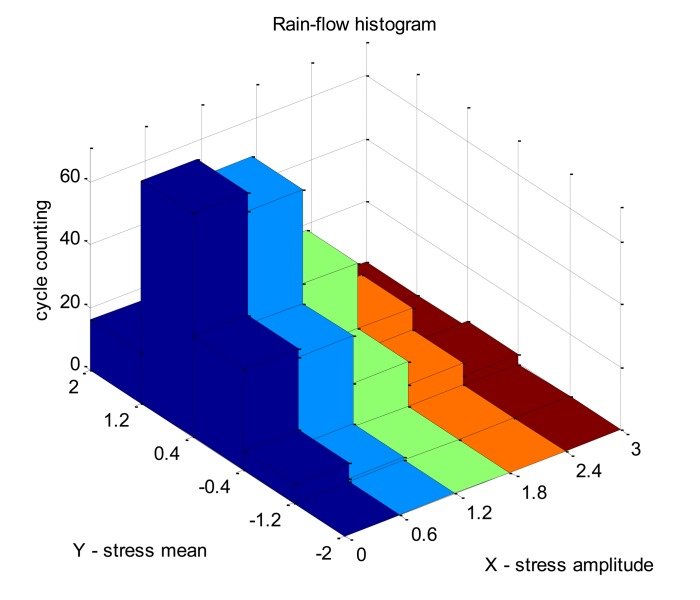
Rain-flow histogram.

To check the feasibility of the system, a numerical simulation was conducted in Matlab. The rain-flow counting method with the four peak-valley counting principle [28] is used in the simulation. The original principle is reprogrammed to remove the invalid amplitudes. Figure 5 shows a group of random signals obtained from the simulation. A total of 637 peak-valley values were detected. Table 1 and Figure 6 show the results from cycle counting. It is noted in Table 1 that the intervals of amplitude and mean values can be set randomly. To get reliable structural fatigue properties, both the theoretical and experimental analysis are expected. The data in Table 1 serves as the basic inputs for counting, and its combination with the material's S-N curve and Miner cumulative damage principle generates the fatigue damage caused by the signals.

**Figure 7.**
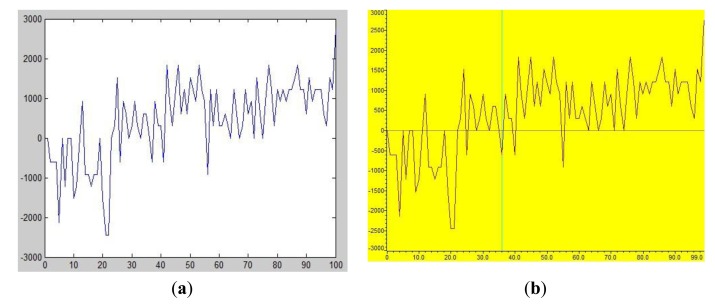
Simulated signal input in Matlab and CCS. (**a**) In Matlab; (**b**) In CCS.

The hardware used in this simulation is a F28335EVM-I development board and the embedded algorithm is programed under the Code Composer Studio (CCS) environment. Figure 7a,b show the simulated signal input in Matlab and CCS, respectively. Tables 2 and 3 list the results of signal outputs from Matlab and CCS. The signals from the Matlab and the CCS show excellent agreement.

### 4.2. Lab Tests Based on Strain Gauge

#### 4.2.1. Material Selection

**Figure 8.**
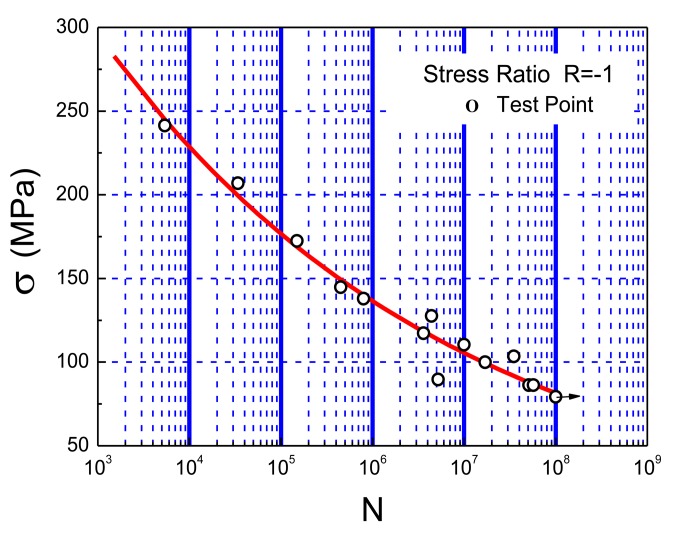
S-N curve of 6061-T6 aluminium alloy.

Lab fatigue tests have been performed to verify the stability, durability, accuracy, and engineering applicability of the developed system. 6061-T6 aluminum alloy, a common material used in mechanical systems, civil infrastructure, automotive, marine structures, and aerospace structures, was selected as the test material due to its high strength, low density, good corrosion resistance, excellent cryogenic toughness, and beautiful appearance. The intrinsic characteristics of aluminum make it sensitive to stress concentration, local damage, and cracks. Table 4 shows the typical chemical elements of 6061-T6 aluminum alloys (% weight) and Figure 8 shows its S-N curve [29].

#### 4.2.2. Specimen Geometric Design

**Figure 9.**
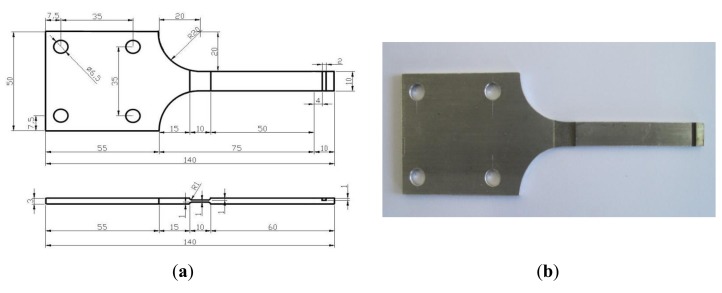
Design of the specimen. (**a**) Geometry parameters of the specimen (unit: mm); and (**b**) Photo of the specimen.

**Figure 10.**
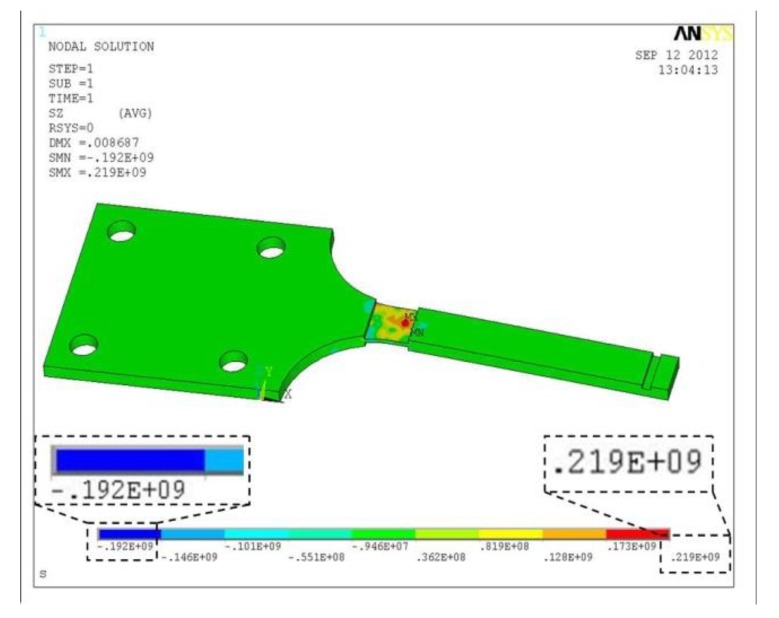
Stress distribution of the specimen.

The geometric design of the specimen mostly depends on the cantilever bending tests. The maximum stress *σ*_max_ of the material is given as follows:

(3) $$\sigma_{\max}=\frac{3E\cdot h\cdot \omega_{B}}{2l^{2}}$$

where *E* is the Youth's modulus, *h* is the height of the specimen, *l* is the distance from the loading point to beam end and *ω_B_* is the maximum loading displacement. When *ω_B_* is 8 mm, *h* is 1 mm, *σ*_max_ is between 75 MPa and 250 MPa, we can get that *l* is from 46.2 mm to 86.4 mm. The gradual geometric change is adopted to the specimen to reduce the residual stress and work-hardening. The gradual geometric change design refers to the Chinese National Standard, “metals-rotating bar bending fatigue testing”. Figure 9 shows the designed geometry layout of the 6061-T6 aluminum alloy specimen. Figure 10 shows the stress distribution of the specimen analyzed by ANSYS with element solid 45 used and the maximum stress is around 219 MPa. Comparison of the computed stress to the S-N curve of 6061-T6 aluminum alloy indicates that the design proposal is feasible.

#### 4.2.3. Experimental Setup

**Figure 11.**
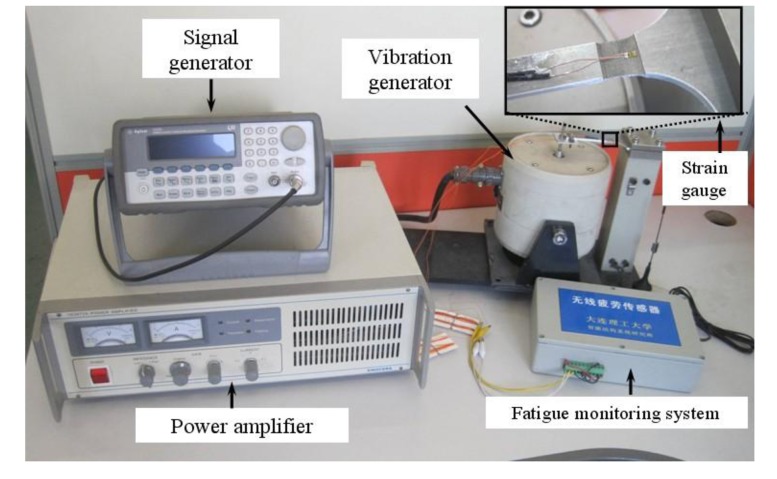
Experimental setup of fatigue test based on strain gauge.

Figure 11 shows the test setup. The fatigue loading is symmetrical, so the stress ratio (R) is −1. Meanwhile, the loading frequency is 5Hz and the power amplifier can control the vibration amplitude. Five specimens were tested under different stress levels.

**Figure 12.**
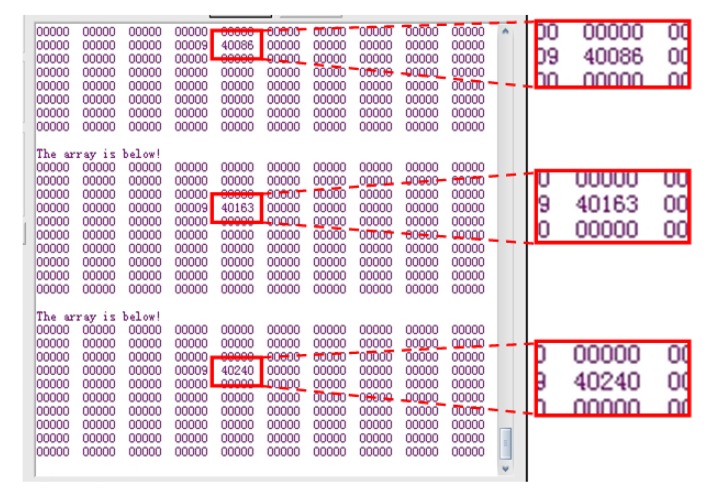
Screenshot of the wireless transceiver software.

The strain gauge served as the sensing probe of the fatigue monitoring system. The rain-flow counting results were sent through wireless transceiver modules and delivered to the PC every 16 seconds. Figure 12 shows a screenshot of the wireless transceiver software.

#### 4.2.4. Results and Discussions

**Figure 13.**
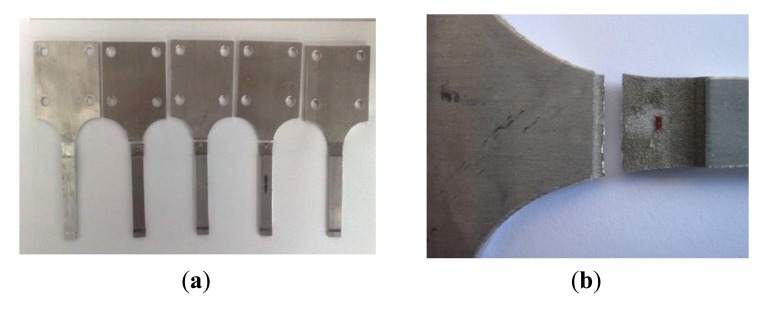
Photos of the damaged specimen. (**a**) Damaged specimen; and (**b**) Fracture morphology.

As shown in Figure 12, the numbers with red frames and enlarged are the three cycle counting results consecutively, which are 40,086, 40,163, and 40,240, increase progressively by 77 every time. The accuracy of the system is high with a relative error less than 4%. With a constant load, only one location on the beam would expect fatigue damage, all other locations would have no response which provide no reading as shown in Figure 12 as zeros. Figure 13a,b show the photos of the damaged specimens. All five specimens indicated the same fatigue failure location as seen in Figure 13a and the fracture is in a serrated shape as shown in Figure 13b, which confirms the lattice restructuring phenomenon of metallic materials under fatigue loading.

**Figure 14.**
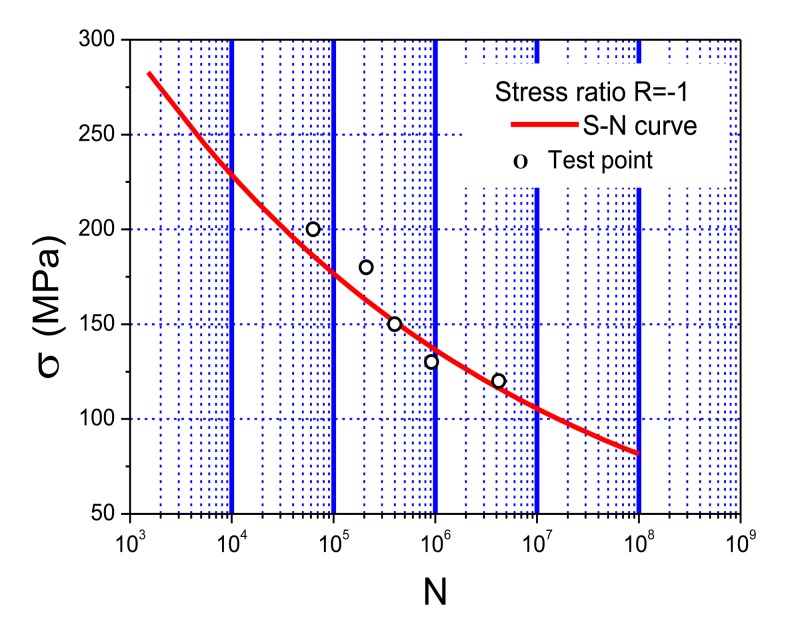
Comparison results of test points and S-N curve.

Table 5 showed the test results of the five specimens. Along with the decrease of stress amplitude, the fatigue life of the specimen apparently increases. The cycle counting of the system is well consistent with the cycles of fatigue loading, and the systematic error is less than 4%, which is caused by omitting cycles during the test. However, it exceeds the accuracy requirement in engineering community. Figure 14 shows the comparison between the test results and the aluminum's S-N curve. Good stability, high accuracy, quick processing, and real-time capability of the fatigue monitoring system was observed during the entire test process. Moreover, the test results also showed that the experimental method is feasible for studying the fatigue properties of 6061-T6 aluminum alloy.

### 4.3. Lab Test Based on the PVDF Piezoelectrc Film

#### 4.3.1. Calibration of the PVDF

The mechanical and the electrical behaviors of PVDF piezoelectric film are expected to be coupled with each other. The piezoelectric equation is used to describe a quantitative relation between its electrical behavior and mechanical behavior. The output of the electric charge from PVDF piezoelectric film is the response of polarization direction caused by strain in all directions. The relationship between the electric charge from the PVDF and the deformation of the monitored structure is calculated by formula (4):

(4) $$V_{0}=\sum g_{3j}\sigma_{j}t$$

**Figure 15.**
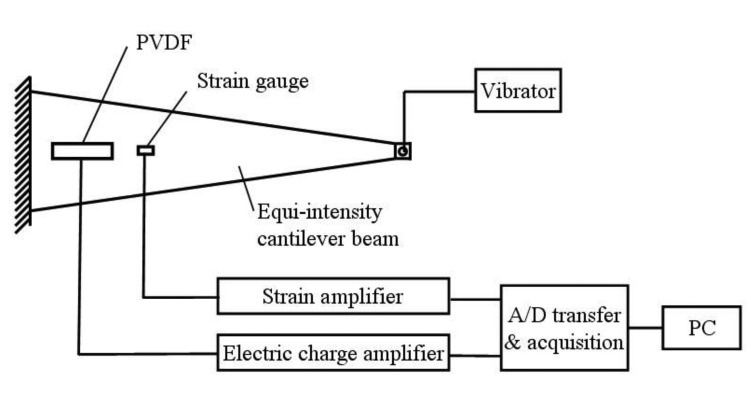
Scheme of sensitivity calibration test of PVDF.

Where *V*_0_ is the PVDF's output voltage, *g*_3_ *_j_* is piezoelectric voltage constant of the PVDF, *σ_j_* is the stress on PVDF, *t* is the thickness of the PVDF, and *j*=1 ∼ 3 Generally, the PVDF's can work in two modes: the stretching mode and the thickness mode. A cantilever beam with a uniform strength, as illustrated in Figure 15, is a flexible structure and the PVDF piezoelectric film only deforms in the direction of tension. Thus, the test beam works in a stretching mode. The output of the PVDF in a stretching mode can be calculated by formula (5):

(5) $$V_{0}=(g_{31}\sigma_{1}+g_{32}\sigma_{2})t$$

As shown in Figure 15, the cantilever beam could be oscillated under the sinusoidal loads. The electrical charge amplifier transfers the signal from the PVDF to the A/D acquisition system, while the dynamic strain amplifier transfers the signal from strain gauge. The A/D transfer and data acquisition system collects and stores both signals from the PVDF and from the strain gauge. The PVDF's sensitivity *K* can be calculated as:

(6) K=ΔV/Δɛ

Where Δ*V* is the output variation of PVDF and Δ*ε* is the output variation of strain gauge.

**Figure 16.**
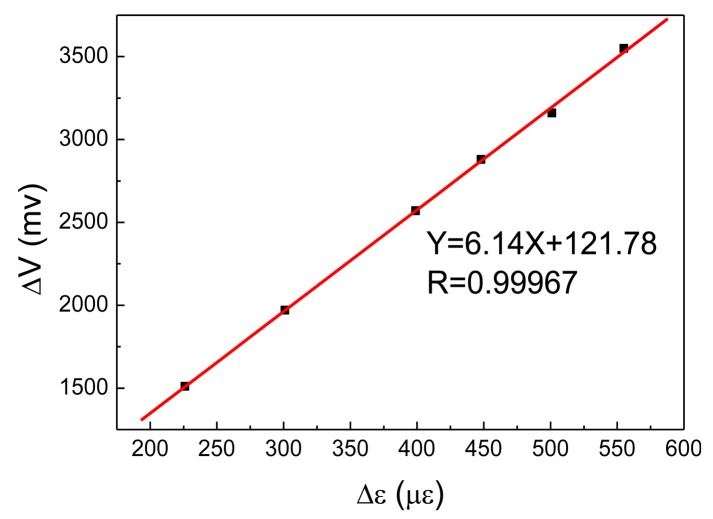
Sensitivity of the PVDF (5 Hz).

Taking 5 Hz loading frequency for example, Δ*V* and Δ*ε* were recorded under different amplitudes, and *K* was calculated and listed in Table 6. With the linear fitting of Δ*V* and Δ*ε*, the sensitivity of the PVDF can be obtained under a loading frequency of 5 Hz. Figure 16 shows corresponding sensitivity of the PVDF, *K* = 6.14 mV/με, and a good linearity was observed.

Similarly, *K* could also be acquired under different loading frequencies, as listed in Table 7, for the PVDF's sensitivities in various frequencies. The sensitivity shows small difference under various loading frequencies.

#### 4.3.2. Experimental Setup

**Figure 17.**
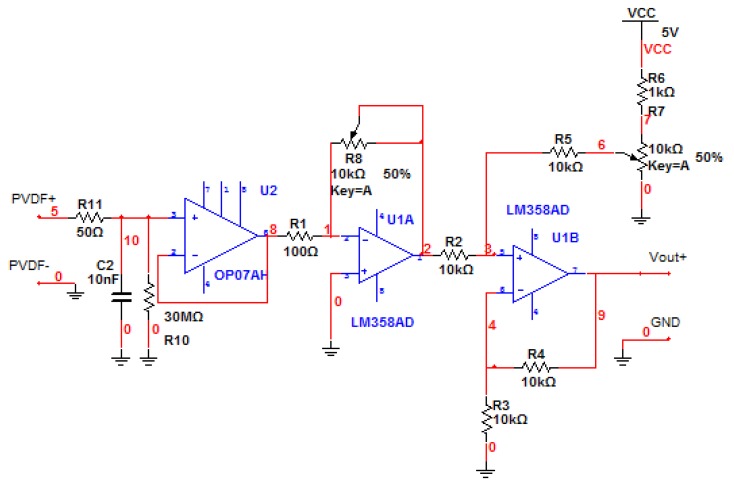
Pre-processing circuit of the unit of the PVDF sensor.

**Figure 18.**
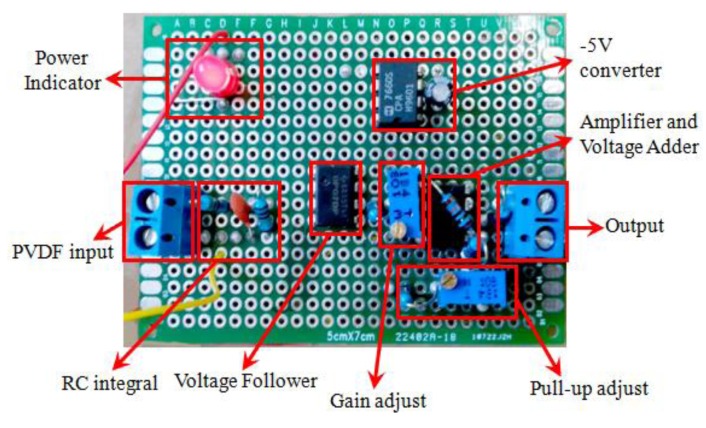
Photo of the pre-processing unit of the PVDF sensor.

An appropriately designed interface circuit plays a key role in the optimization of the PVDF sensors. The signal output of the PVDF sensor is expected to be only ±10–20 mv, meanwhile, the signal has a high output resistance and a high Gaussian noise, which contributes to the inaccuracies of the sampling process. Considering the frequency range and signal amplitude requirements over the desired dynamic range for the sampling circuit, the voltage of output signal needs to be maintained in the range of 0–3 V. The most critical part of an interface circuit is a proper input resistance. The input resistance affects low frequency measurement capability as well as signal amplitude, which is called the “loading effect”. To meet the needs of high sampling rate and to improve signal integrity, a well-designed interface circuit is introduced into the system as shown in Figure 17, including: (1) a RC integral circuit; (2) a voltage follower; (3) a voltage amplifier; (4) a voltage generator; and (5) a voltage adder. A RC integral circuit converts the charges generated by the PVDF into voltage signal. Figure 18 is a photogragh of the unit. A voltage follower reduces the loading effect and isolates the input from the output. A voltage amplifier offers an adjustable gain ranged from 1 to 100, facilitating a desired output for sampling purpose. Regarding to the dynamic range requirements, a voltage adder pulls the signal above the ground. By applying the basic circuit principles, the output voltage, *V*_0_, can be calculated as:

(7) $$V_{0}=-\frac{R_{8}}{R_{1}}V_{\mathrm{PVDF}+}+\frac{R_{6}}{R_{6}+R_{7}}V_{+5V}$$

Where *R*_8_ is the gain resistance, *R*_7_ regulates the output pull-up, and *V_PVDF_*_+_ is the input of PVDF.

**Figure 19.**
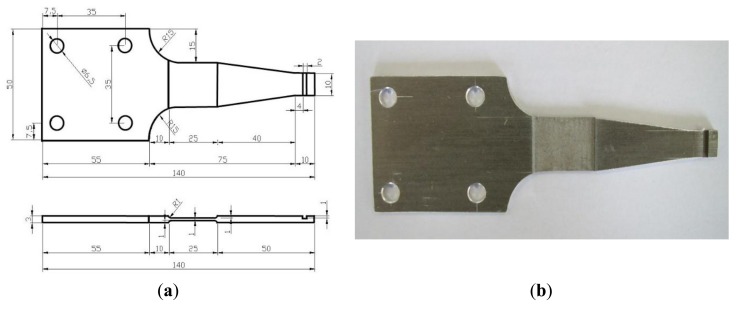
Design of the specimen. (**a**) Geometry parameters of the specimen; and (**b**) Photo of the specimen.

**Figure 20.**
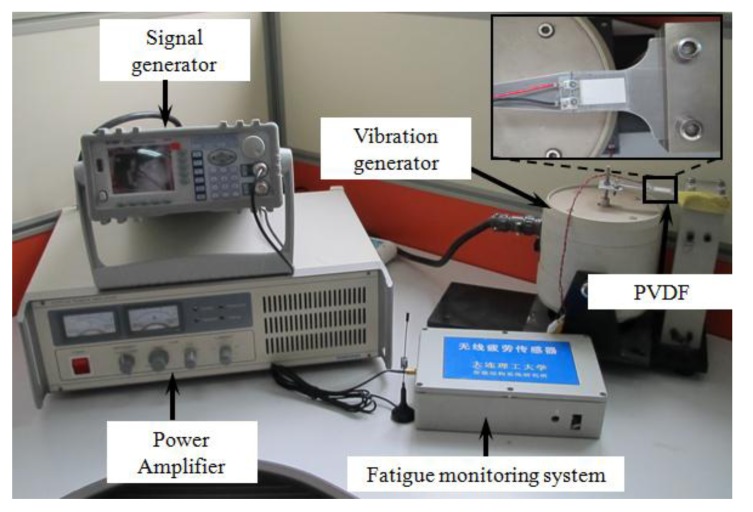
Experimental setups of fatigue tests based on PVDF sensors.

6061-T6 aluminum alloy is used again as the test material in this lab test. The geometric layout of the test specimen is illustrated in Figure 19, with the test setup shown in Figure 20. Again, the symmetrical stress ratio equals to negative 1 because of fatigue loading, so, *R* = −1 Meanwhile, the frequency of the load is 10 Hz and a power amplifier is used to control the vibration amplitude. Nine specimens were tested under different stress level.

**Figure 21.**
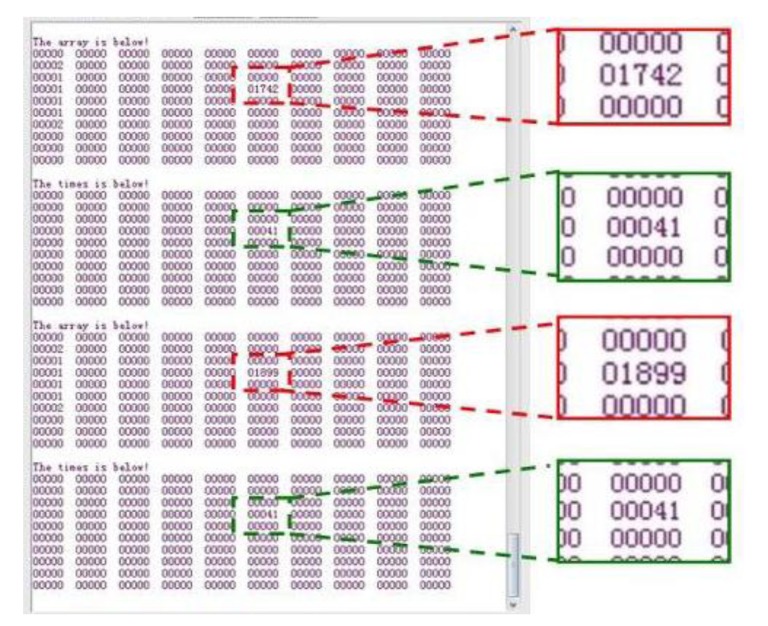
Screenshot of the wireless transceiver software.

Figure 21 shows a screenshot of the wireless transceiver software of the PVDF sensor tests. The numbers enlarged and framed in red are the consecutively increased counting results of two cycles, which are 1,742 and 1,899. The enlarged numbers in the green frames will increase by one once the cycle counting is cumulative to 10,000. For example, the number in Figure 21 is 41, which means the cycle counting is 410,000. The accuracy of the system is high, with a relative error of less than 5%.

#### 4.3.3. Test Results and Discussions

**Figure 22.**
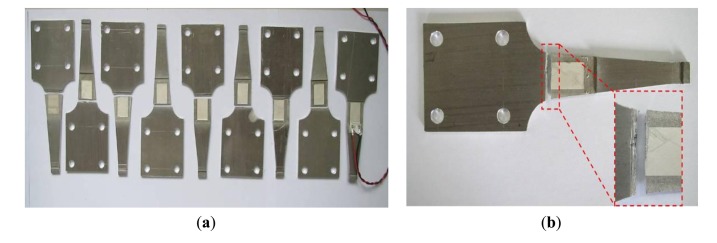
Photos of the damaged specimen. (**a**) Damaged specimen; and (**b**) Fracture morphology.

Figures 22a,b show photos of the damaged specimens. All the specimens were broken at nearly the same location. The fracture surface was also in a serrated shape, which confirms the lattice restructuring phenomenon of metallic materials under fatigue loading, as stated in Section 4.2.1 above.

**Figure 23.**
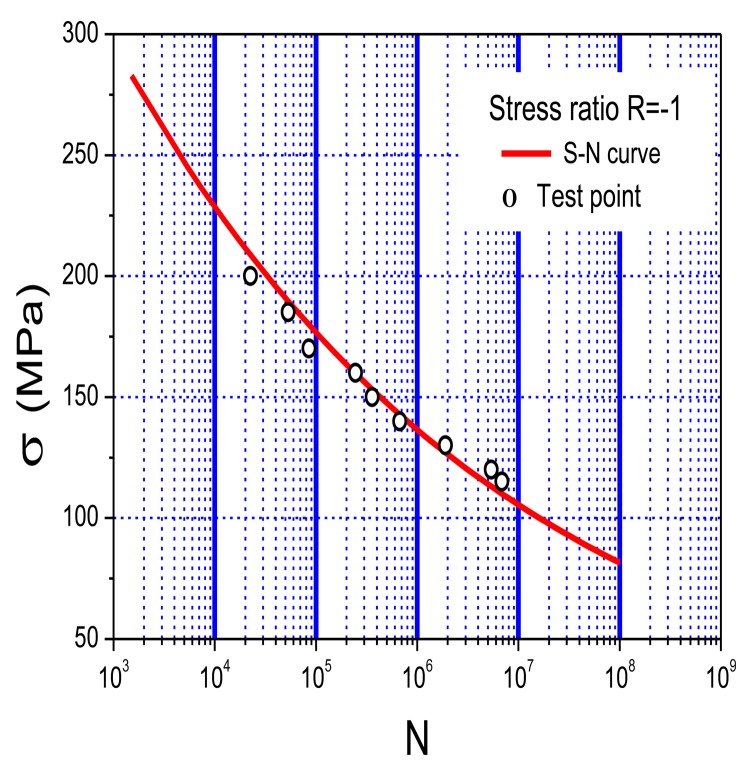
Comparison results of test results and the S-N curve.

Table 8 illustrates the test results of the nine specimens. With the decrease of stress amplitude, the fatigue life of the specimen apparently increases. The systematic error is less than 5%, which is mostly caused by omitting cycles during the test but meets the requirement in engineering community. Figure 23 shows the comparison between the test results and the S-N curve of the material. The results not only show the excellent properties of the system based on the PVDF such as good stability, high accuracy, quick processing, and real-time capacity, but also verify the test results of the system based on the strain gauge.

## 5. Conclusions

In this paper, a novel wireless fatigue monitoring system based on the bio-inspired tree-ring data tracking method, was designed, investigated, and verified through theoretical, numerical, and experimental studies. Two types of sensing probe, the strain gauge and the PVDF sensors were utilized for the validation of the developed system. Laboratory fatigue tests of the system based on the strain gauge and the PVDF sensors showed a relative systematic error of less than 5%, which can satisfy the accuracy requirements of the engineering community. The test results showed that the developed system possessed good stability, high accuracy, fast processing, and real-time capability. Thus, the developed system is promising to be further applied in fatigue damage accumulation analysis and early warning for potential fatigue related structure failures in practice.

## Figures and Tables

**Table 1.** Rain-flow matrix for cycle counting.

**Amplitude**	**[0, 0.6]**	**[0.6, 1.2]**	**[1.2, 1.8]**	**[1.8, 2.4]**	**[2.4, 3]**

**Mean**					
[−2, −1.2]	0	0	0	0	0
[−1.2, 0.4]	6	1	0	0	0
[−0.4, 0.4]	32	29	14	7	3
[0.4, 1.2]	71	65	35	14	1
[1.2, 2]	16	10	5	1	1

**Table 2.** Result of signal output in Matlab.

	**1**	**2**	**3**	**4**	**5**	**6**	**7**	**8**	**9**	**10**
1	2	1	0	0	0	0	0	0	0	1
2	0	0	0	0	0	0	0	0	0	0
3	0	0	0	0	0	0	0	0	0	0
4	0	0	0	0	0	0	0	0	0	0
5	0	2	0	0	0	0	0	0	0	0
6	0	0	0	0	0	0	0	0	0	0
7	3	0	0	0	0	1	0	0	1	0
8	0	1	0	0	0	0	0	0	0	0
9	0	0	0	0	0	0	0	0	0	0
10	1	3	0	0	0	0	0	0	0	0

**Table 3.** Result of signal output in CCS.

	**1**	**2**	**3**	**4**	**5**	**6**	**7**	**8**	**9**	**10**
1	0×002	0×001	0×000	0×000	0×000	0×000	0×000	0×000	0×000	0×001
2	0×000	0×000	0×000	0×000	0×000	0×000	0×000	0×000	0×000	0×000
3	0×000	0×000	0×000	0×000	0×000	0×000	0×000	0×000	0×000	0×000
4	0×000	0×000	0×000	0×000	0×000	0×000	0×000	0×000	0×000	0×000
5	0×000	0×002	0×000	0×000	0×000	0×000	0×000	0×000	0×000	0×000
6	0×000	0×000	0×000	0×000	0×000	0×000	0×000	0×000	0×000	0×000
7	0×003	0×000	0×000	0×000	0×000	0×001	0×000	0×000	0×001	0×000
8	0×000	0×001	0×000	0×000	0×000	0×000	0×000	0×000	0×000	0×000
9	0×000	0×000	0×000	0×000	0×000	0×000	0×000	0×000	0×000	0×000
10	0×001	0×003	0×000	0×000	0×000	0×000	0×000	0×000	0×000	0×000

**Table 4.** Typical chemical compositions of 6061-T6 aluminium alloys (% weight) [30,31].

	**Al**	**Cr**	**Cu**	**Fe**	**Mg**	**Mn**	**Si**	**Ti**	**Zn**
Minimum	95.8	0.04	0.15	-	0.8	-	0.4	-	-
Maximum	98.6	0.35	0.4	0.7	1.2	0.15	0.8	0.15	0.25

**Table 5.** Fatigue test result based on strain gauge.

**Specimen No.**	**Stress amplitude**	**Cycles of loading**	**Cycles of system**	**Error**	**Fatigue life**	**Remark**
1	200 mPa	63,000	60,500	4%	33,700	Fracture
2	180 mPa	210,000	202,000	3.8%	79,100	Fracture
3	150 mPa	400,000	389,600	2.6%	432,000	Fracture
4	130 mPa	918,000	888,300	3.2%	1,550,000	Fracture
5	120 mPa	4,200,000	4,051,100	3.5%	3,100,000	Not broken

**Table 6.** The output of PVDF and strain gauge (5 Hz).

**ΔV_PVDF_/mV**	**Δε_strain_/με**	**K(ΔV/Δε)**
1510	226	6.68
1970	301	6.54
2570	399	6.44
2880	448	6.43
3160	501	6.31
3550	555	6.40

**Table 7.** *K* under different loading frequencies (0.5∼20 Hz).

**Loading frequencies (Hz)**	*K* **(***mv* / *με***)**
0.5	5.71
1	6.45
2	6.34
5	6.14
10	6.68
15	7.38
20	7.88

**Table 8.** Fatigue test results based on the PVDF.

**Specimen No.**	**Stress amplitude**	**Cycles of loading**	**Cycles of system**	**Error**	**Fatigue life**	**Remark**
1	200 mPa	22,800	22,500	1.15%	33,700	Fracture
2	185 mPa	54,300	53,236	2%	65,700	Fracture
3	170 mPa	87,600	85,300	2.6%	141,000	Fracture
4	160 mPa	258,000	245,800	4.72%	243,000	Fracture
5	150 mPa	365,400	358,000	2.03%	432,000	Fracture
6	140 mPa	688,200	671,700	2.4%	799,000	Fracture
7	130 mPa	1,964,400	1,908,500	2.85%	1,550,000	Fracture
8	120 mPa	5,485,200	5,390,300	1.73%	3,100,000	Fracture
9	115 mPa	7,000,000	6,874,400	1.79%	4,660,000	Not broken
